# Challenges in extracting a defective ventricular lead after CRT-D: a case report

**DOI:** 10.3389/fcvm.2024.1464620

**Published:** 2025-01-10

**Authors:** Murat Özmen, Faruk Aydınyılmaz

**Affiliations:** Department of Cardiology, University of Health Sciences, Erzurum Bolge Training and Research Hospital, Erzurum, Turkey

**Keywords:** coronary sinus lead, rupture, pacemaker, complication, to manage

## Abstract

**Background:**

Our aim in this case was to remove the defective ventricular lead and the ruptured coronary sinus lead.

**Methods:**

Entering through the right femoral vein and removing the coronary sinus lead with a pigtail catheter.

**Results:**

In our attempt to extract the coronary sinus lead, it fractured. The broken fragment was successfully removed without any complications.

**Conclusions:**

In this case, which is very rarely encountered in daily practice, we successfully removed the coronary sinus lead from the body from the femoral vein using a pigtail catheter with a new method.

## 1 Introduction

Pacemakers (PM) are well-known devices for the treatment of bradyarrhythmias and play an important role in saving the lives of more than a million people worldwide every year. Both clinical practice and numerous studies have objectively proven pacemakers' effectiveness in terms of patient quality of life, morbidity, and mortality. Undoubtedly, relevant technologies have made great leaps during the same period (1). Today, thanks to developments in microelectronics, devices have become smaller, programming options have increased, and pacemaker leads have become thinner and longer-lasting. However, despite continuous technological advances, pacemakers are still associated with significant complications. These complications are mostly lead or pocket-related. Immediate and short-term complication rates can be as high as 12%. These short-term complications mainly occur as pocket hematoma, pneumothorax, cardiac tamponade, and lead dislodgement (2). Long-term complications include tricuspid regurgitation, venous obstruction, lead fractures, insulation failure, and device-related infection. The incidence of long-term complications is 9%, and lead-related endocarditis is associated with a higher risk of mortality, ranging from 12% to 31%. Lead fractures, usually caused by weight lifting or chest trauma, are characterized by damage to one or more pacemaker electrodes (3). In addition, extending the life of devices and eliminating major and minor complications related to treatment have become indispensable goals of both manufacturers and physicians. Over the last 12 years, there has been further progress in electrical stimulation and entry into the field of ventricular resynchronization as adjunctive therapy for patients with drug-refractory heart failure and delayed ventricular conduction.

## 2 Case report

**Figure 1:**
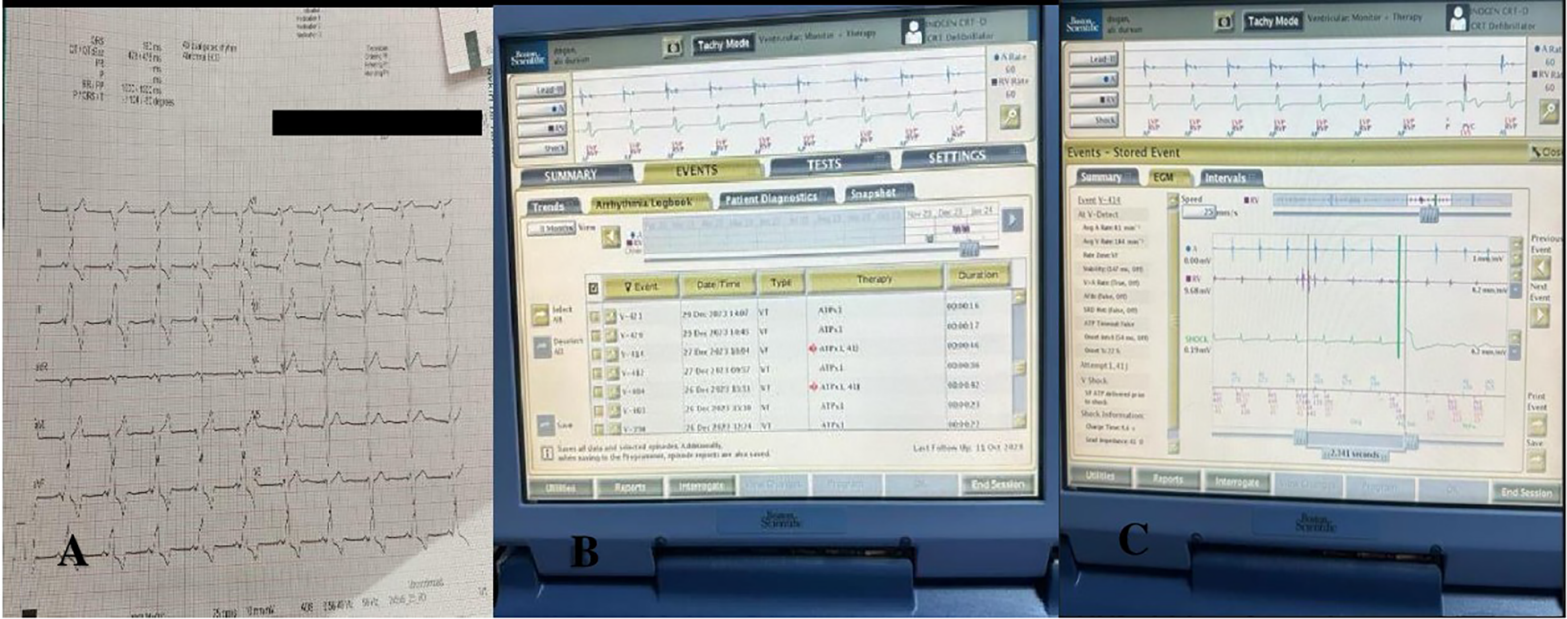
**(A)** ECG, **(B)** repeated battery shocking, **(C)** microfracture.

A 65-year-old patient with heart failure, hypertension, and pacemaker shock who had undergone CRT-D implantation 3 years ago was admitted to the emergency department. The patient was hospitalized at our clinic. Blood pressure was measured as 130/70 in the right arm, 120/70 in the left arm, pulse 80/min, and saturation 90. No abnormal condition was detected in the patient's electrocardiography (ECG) (Figure 1A). Echocardiographic examination revealed an ejection fraction of 30%, mild-to-moderate mitral regurgitation, systolic pulmonary artery pressure of 42mmHg, and an appearance compatible with the pacemaker lead in the right heart. No vegetation or abscess was observed. Battery measurements of the patient were made. The battery measurements showed that the patient received frequent shocks (Figure 1B). In repeated measurements, a microfracture was detected in the right ventricular lead (Figure 1C).

**Figure 2:**
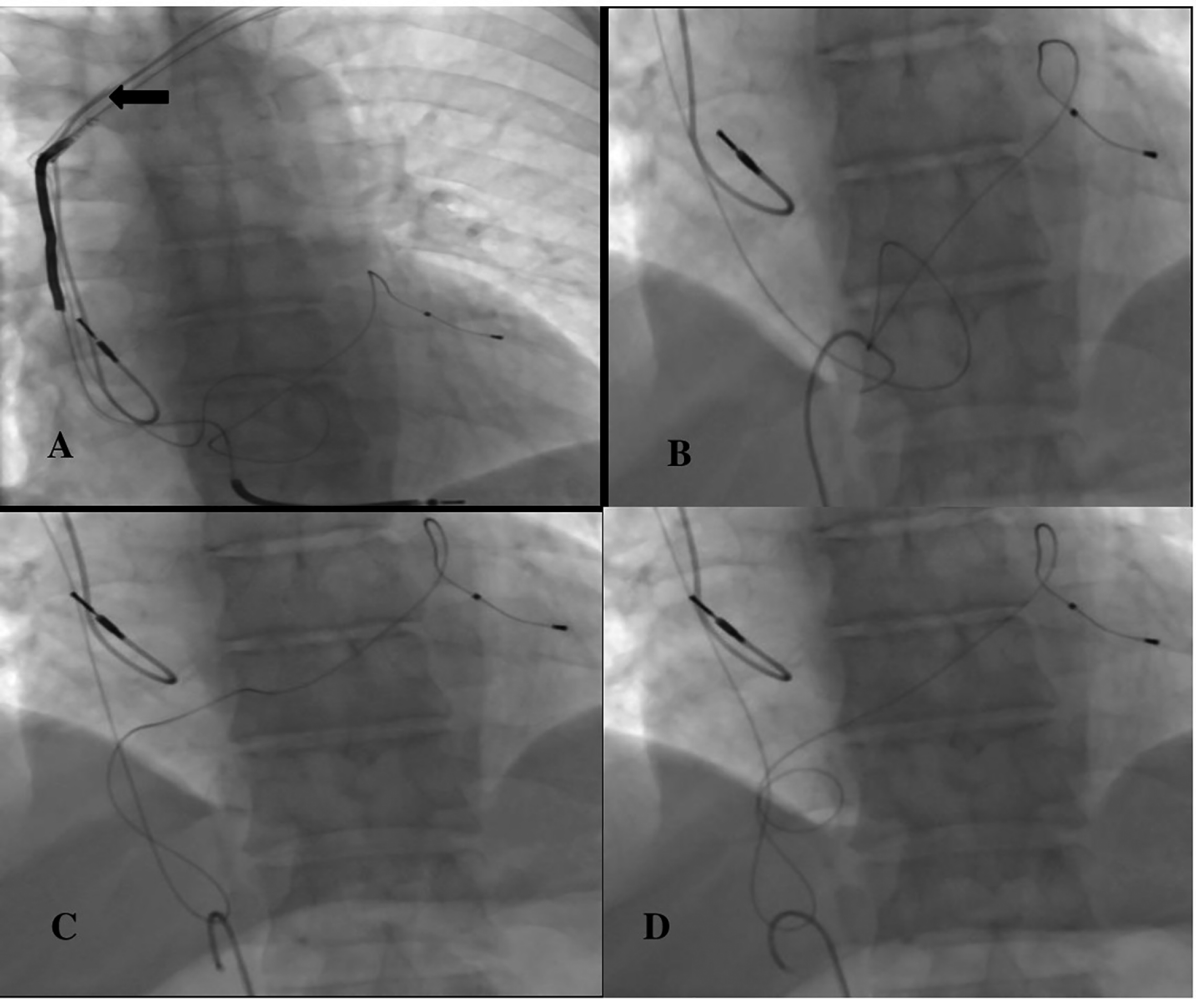
**(A)** ← Broken CS lead, **(B)** capturing the CS lead with a pigtail catheter, **(C,D)** turning the pigtail catheter clockwise and wrapping it around the lead.

**Figure 3:**
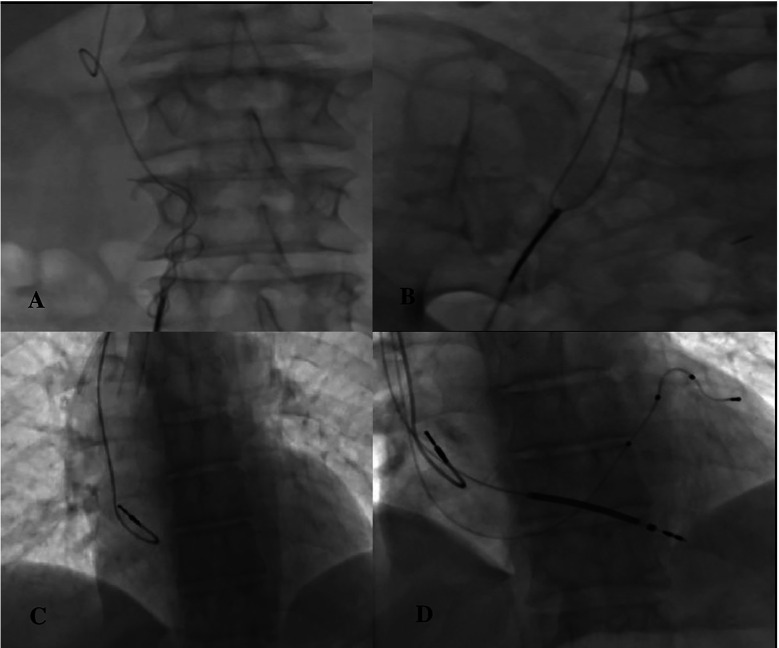
**(A)** Withdrawing the coiled lead, **(B)** capturing the lead separated from the pigtail with a snare, **(C)** completely removing the ventricle and CS lead, **(D)** reimplantation.

The patient was taken to the cath laboratory, and an incision was made at the pace site, and the leads were accessed. The transvenous lead extraction device (TLED) was advanced towards the superior vena cava, incorporating the right ventricular lead. Meanwhile, there was difficulty in moving the device forward. Subsequently, the coronary sinus (CS) lead broke off. (Figure 2A). The process continued. First, the right ventricular lead was removed. Then, the 9F sheath was entered through the right femoral vein. A 6F pigtail catheter was sent from the sheath. An attempt was made to capture the CS lead with the catheter. The tip of the catheter caught the lead, (Figure 2B) it was turned clockwise and wrapped around the catheter in a spiral shape (Figure 2C). By applying a downward pulling force, it was seen that the CS lead was coming (Figure 2D). The lead was withdrawn up to the femoral sheath (Figure 3A). After withdrawing up to the femoral sheath, it was observed that the lead was stripped from the catheter again at the entrance of the sheath. The lead was captured and removed by sending a snare through the sheath (Figure 3B). No effusion was observed on echocardiography. The procedure was terminated by reinserting the ventricle and coronary sinus lead (Figures 3C,D). The procedure took approximately 25 min from the breakage of the Cs lead until it was removed with a pigtail. During this process, 300 gray of radiation was exposed. After the patient was monitored in the coronary intensive care unit for 1 day, he was transferred to the ward and discharged. No complications or problems were observed during the 1st and 3rd month follow-up.

## 3 Discussion

Pacemakers play an important role in the treatment and management of patients with bradyarrhythmia and heart failure. However, post-implantation complications can lead to significantly worse outcomes (4).

A study reported that lead fractures may be caused by subclavian syndrome (5). However, in our study, the existing lead fractures were not due to this situation. Damage from subclavian crush syndrome is a gradual process. Additionally, this syndrome should damage all leads. In our patient, there was only a problem with the ventricular lead. The atrium and coronary sinus leads were still working. In another study, the broken lead was not removed from the body to avoid complications (6). In our case, the coronary sinus lead was removed successfully, and no complications were observed. In another study, the CS lead fracture could not be removed from the body despite surgical and vascular procedures, and a patch was applied to the terminal lead area (7). In our case, we successfully removed the broken lead from the femoral vein. In another study, an attempt was made to remove the broken lead from the femoral vein with a snare, but it was unsuccessful (8). In our case, the snare was used at the last stage and was successful.

In our case, the use of snare was considered as an alternative. However, a pigtail was decided since one of the leads’ ends was in the vena cava and the other was in the coronary sinus. The decision potentially carried the risk of entanglement in the right atrium lead. If it could not be removed with a pigtail, the broken lead could be left in that area, and a new lead could be inserted next to it. Our case has not been seen before in the literature, and this complication was successfully managed by rupture of the coronary sinus lead while the ventricle lead was retrieved during the procedure and was successfully removed from the femoral vein with a pigtail catheter.

Clinicians who encounter such similar cases may consider capturing the broken piece after the complication with a snare from inside the atrium. If it cannot be removed, an alternative approach may be to send a second coronary sinus lead next to it if the ruptured segment is within the coronary sinus and the coronary sinus diameter is suitable. As a result, our case may help the clinician in resolving a rare complication with different approaches. It may be an alternative to rare approaches in the literature on this subject. It should be kept in mind that, in this case, the broken lead can be removed via the femoral vein. This case report is the first documentation of successful pigtail removal of a fractured coronary sinus lead.

## Data Availability

The raw data supporting the conclusions of this article will be made available by the authors, without undue reservation.

## References

[1] Mond HG Proclemer A. The 11th world survey of cardiac pacing and implantable cardioverter-defibrillators: calendar year 2009-a world society of arrhythmia’s project. Pacing Clin Electrophysiol. (2011) 34:1013–27. 10.1111/j.1540-8159.2011.03150.x 21707667

[2] Kirkfeldt RE Johansen JB Nohr EA Jorgensen OD Nielsen JC. Complications after cardiac implantable electronic device implantations: an analysis of a complete, nationwide cohort in Denmark. Eur Heart J. (2014) 35:1186–94. 10.1093/eurheartj/eht511 24347317 PMC4012708

[3] Udo EO Zuithoff NPA van Hemel NM de Cock CC Hendriks T Doevendans PA Incidence and predictors of short- and long-term complications in pacemaker therapy: the FOLLOWPACE study. Heart Rhythm. (2012) 9:728–35. 10.1016/j.hrthm.2011.12.014 22182495

[4] Khattak F Khalid M Gaddam S Ramu V Brahmbhatt V. A rare case of complete fragmentation of pacemaker lead after a highvelocity theme park ride. Case Rep Cardiol. (2018) 2018:4192964. 10.1155/2018/4192964 30498603 PMC6220749

[5] Kotsakou M Kioumis I Lazaridis G Pitsiou G Lampaki S Papaiwannou A Pacemaker insertion. Ann Transl Med. (2015) 3(3):42. 10.3978/j.issn.2305-5839.2015.02.06 25815303 PMC4356861

[6] Kusumoto FM Schoenfeld MH Willkoff BL Berul CI Birgersdotter-Green UM Carrillo R 2017 HRS expert consensus statement on cardiovascular implantable electronic device lead management and extraction. Heart Rhythm. (2017) 14(12):e503–51. 10.1016/j.hrthm.2017.09.001 28919379

[7] Gardini A Fracassi F Saporetti A Mariggio D. Reconstruction of the terminal of an abandoned fractured unipolar coronary sinus lead: a feasible solution to restore effective cardiac resynchronization therapy. Indian Pacing Electrophysiol J. (2013) 13(3):122–5. 10.1016/S0972-6292(16)30630-1 23840107 PMC3691391

[8] Bontempi L Aboelhassan M Cerini M Salghetti F Arabia G Fabbricatore D Abandoned and fractured ICD lead with complete superior veins occlusion: is transvenous lead extraction still possible? J Cardiovasc Electrophysiol. (2020) 31(11):3042–4. 10.1111/jce.14752 32955129

